# Methyl jasmonate counteracts cadmium toxicity in water spinach plant by adjusting growth, physiology and redox regulation

**DOI:** 10.1038/s41598-025-09852-9

**Published:** 2025-07-10

**Authors:** Md. Sabibul Haque, Shitosri Mondal, Kh Sabbir Hossain, Artho Baroi, Md. Tanveer Hussain, Md. Ashik Mia, Md. Nesar Uddin, A. K. M. Golam Sarwar, Md. Alamgir Hossain, Md Amirul Islam

**Affiliations:** 1 Department of Crop Botany, Bangladesh Agricultural University, Mymensingh, 2202 Bangladesh; 2 Department of Plant Nutrition, Institute of Crop Science and Resource Conservation, University of Bonn, Bonn, Germany

**Keywords:** Antioxidants, Cd tolerance, Gas exchange, Jasmonate, Leafy vegetable, Oxidative stress, Plant sciences, Plant hormones, Plant physiology, Plant stress responses

## Abstract

Increased cadmium (Cd) level in foods due to anthropogenic activities is a serious concern to public health. This study investigated the efficacy of exogenous methyl jasmonate (MeJA) application to mitigate adverse effects of Cd toxicity in water spinach plant. The seeds (cv. Gimakolmi) were primed with MeJA (2.5 and 5 µM) and grown under two levels of Cd (10 and 20 µM CdCl_2_) with or without the respected levels of MeJA solutions under the hydroponic system. The experiment was set in a completely randomized design with three replications maintaining seven growth conditions: (1) Control, (2) Cd_10_, (3) Cd_20_, (4) Cd_10_MJ_2.5_, (5) Cd_10_MJ_5_, (6) Cd_20_MJ_2.5_ and (7) Cd_20_MJ_5_. Cd-stress significantly hindered growth and photosynthesis; induced oxidative damage accumulating higher malondialdehyde (MDA) and H_2_O_2_ contents; enhanced activities of antioxidative enzymes and increased Cd uptake in water spinach plant. The treatments Cd_10_MJ_5_ and Cd_20_MJ_5_ stimulated plant growth by increasing total dry mass (66% and 38%) and rate of photosynthesis (51% and 55%) of water spinach under two levels of Cd stress, respectively. Application of 5 µM MeJA considerably reduced leaf MDA (32% and 17% compared to Cd_10_ and Cd_20_, respectively) and H_2_O_2_ contents (49 and 42%) and enhanced the activities of superoxide dismutase (71% and 6%), catalase (120% and 61%) and peroxidase (57% and 65%) enzymes with reduced uptake of total Cd (38% and 45%) in water spinach plant. Conclusively, 5 µM MeJA effectively mitigated Cd toxicity in water spinach plant and can be adopted in Cd-contaminated areas with further field trials.

## Introduction

Cadmium (Cd) is a highly toxic and mobile heavy metal, and even at low concentrations, it can be hazardous to all living organisms^1^. Injudicious disposal of industrial and domestic waste, along with the indiscriminate use of agrochemicals, mining, and sewage sludge, contributes to Cd accumulation in agricultural soils and groundwater, making it available to the plants^2^. Cadmium contamination is a global concern, with Cd levels in agricultural soils ranging from 0.11 to 5.20 mg/kg across various regions^3^. In Bangladesh, the cultivated soils and rivers adjacent to highways and industrial areas particularly in Dhaka, Narayanganj, Gazipur and Mymensingh districts including different export processing zones (EPZs) displayed much higher Cd than the acceptable limits^4–6^. Cd levels in most vegetables growing in these areas exceed the recommended values possessing potential health risk to human^7,8^. Water spinach (*Ipomoea aquatica* Forsk.) is a very popular leafy vegetable in Bangladesh because it is cheaper than other leafy vegetables and can be cultivated easily in amphibious conditions. This plant has the higher ability to uptake Cd through roots from Cd-polluted soils and rapidly translocate Cd from root to the edible leaves of the plant^9,10^. Cd levels in water spinach leaves ranged from 0.17 to 0.83 mg/kg (dry weight basis) nearby to industrial areas in Bangladesh which were higher than that of agricultural soil and exceeded the permissible limit recommended by the World Health Organization and Food and Agriculture Organization^10,11^. Leafy vegetables are known as Cd-accumulators due to their extent potential of Cd uptake and translocation, and as a key diet, play a major pathway of Cd exposure to the human body^12–14^. Abundant Cd within the plant affects diverse morpho-physiological and biochemical attributes inhibiting photosynthesis, chlorophyll production, growth, biomass and yield of crop plants^15–20^. Cd stress causes stomatal closure and impairs photosystems I and II particularly the light-harvesting system II leading to restriction of photosynthesis^20–22^. Increased Cd in the soil impairs nutrient uptake and water absorption, affecting root and shoot elongation, respiration, and other metabolic pathways^23,24^. Cd toxicity induces oxidative damage by significantly increasing reactive oxygen species (ROS) production^25,26^. To counteract this, plants have developed defense mechanisms, including enzymatic antioxidants, such as superoxide dismutase (SOD), peroxidase (POD), ascorbate peroxidase (APX) and catalase (CAT), as well as nonenzymatic scavengers, including glutathione, carotenoids, and ascorbate^16,27^.

Various approaches have been employed to mitigate the toxicity, uptake and translocation of Cd in vegetables. These include the use of both organic and inorganic amendments, such as silicon, compost, biochar and manure^28^ as well as the exogenous application of nanoparticles^18,24^ and phytohormones^29,30^. Phytohormones, which are active even at low concentrations^31^ play a crucial role in the plant’s adaptation and defense against heavy metal toxicity^17,30^. Additionally, the plant’s response to Cd stress involves the enhanced synthesis of certain signaling molecules, particularly phytohormones such as salicylic acid (SA), jasmonic acid (JA), ethylene (ET) and methyl jasmonate (MeJA)^32^. Jasmonates (JAs), which include JA and MeJA, are a class of cyclopentanone compounds formed through the octadecanoic pathway from linolenic acid. Generally, MeJA alters several physiological and biochemical descriptors in plants improving growth, accumulation of active compounds and endogenous hormones levels under abiotic stress^33,34^. Extensive studies indicate that MeJA increases plant dry weight, leaf chlorophyll content and osmolyte concentrations; upregulates antioxidant activities (SOD, APX, CAT and POD); and scavenges ROS, thereby improving plant resistance to oxidative damage under heavy metal stress^35–39^. MeJA protects plants from heavy metal toxicity by enhancing secondary metabolites production and the expression of stress resistance genes^33,35^. The exogenous application of MeJA has been reported as an effective approach to reduce Cd uptake and alleviate the negative effects of Cd toxicity in various crops such as tomato^40^; rice^41^; okra^42^; mustard^43^; hot pepper^44^; soybean^45^; *Kandelia obovata*^46^; pea^47^ and wheat^48^.

In Bangladesh, potential phytohormone such as MeJA application for the attenuation of Cd-toxicity in leafy vegetables has not yet been practically applied or documented. The exogenous application of phytohormones is entirely dose- and species-dependent and therefore, optimization of hormonal levels under Cd stress and comprehensive understanding of the morpho-physiological and biochemical responses of MeJA to Cd stress at whole plant levels are crucial. This study aimed to optimize the concentration of MeJA for exogenous application in water spinach plant and to evaluate the potential role of MeJA in mitigating Cd-toxicity by modulating metal uptake, growth, physiology and redox homeostasis. The study hypothesizes that the exogenous application of MeJA to water spinach will reduce Cd uptake and translocation in plant tissues while enhancing plant growth, physiological and biochemical traits and stress resistance, offering insights into its broader applicability in the production of leafy vegetables in Cd-contaminated soils of Bangladesh.

## Materials and methods

### Plant materials and growth conditions

An experiment was set in the hydroponic chamber of the Department of Crop Botany, Bangladesh Agricultural University, Mymensingh, Bangladesh from August 2023 to December 2023. A popular cultivar of water spinach (Gimakolmi) was collected from BRAC Seed and Agro Enterprise, Dhaka, Bangladesh and the seeds were disinfected with Vitavax-200 @ 1.5 g kg^−1^ seed. The seeds were primed with distilled water for control and Cd alone treatments (10 and 20 µM CdCl_2_·H_2_O) and with two concentrations of methyl jasmonate (MeJA; 2.5 and 5 μM) for growing in cadmium stress with MeJA conditions. The applied Cd and MeJA concentrations were chosen from a series of prior trials along with comprehensive literature review^23,30^. The ratio of seed weight to priming solution volume (w/v) was maintained at 1:6 and priming was executed for 12 h at 25 °C with proper aeration under dark conditions. Then the seeds were properly rinsed and dried at room temperature to acquire the initial moisture level. To facilitate germination, the primed seeds were placed into a net keeping in gentle touch with water behind the net with the help of a pot filled with aerated water. A total of seven growth combinations were established under hydroponic system: (1) Control (2) Cd_10_ (3) Cd_20_ (4) Cd_10_MJ_2.5_ (5) Cd_10_MJ_5_ (6) Cd_20_MJ_2.5_ and (7) Cd_20_MJ_5_. Rectangular plastic tanks (L × W × H; 11″ × 7″ × 7″) were used for hydroponic culture using perforated cork sheets with 14 holes as trays. Pregerminated 6-day-old water spinach seedlings were inserted into the holes with the help of styrofoam. Each tank was filled initially with 2.5 L of modified half strength nutrient solution having a pH ranging from 6 to 6.5 with the following nutrient composition^49^: Ca(NO_3_)_2_·4H_2_O (2.5 mM), K_2_SO_4_ (1 mM), KH_2_PO_4_ (0.2 mM), MgSO_4_·7H_2_O (0.5 mM), CaCl_2_·2H_2_O (2 mM), H_3_BO_3_ (1 μM), MnSO_4_·6H_2_O (2 μM), ZnSO_4_·7H_2_O (0.5 μM), CuSO_4_·5H_2_O (0.3 μM), (NH_4_)_6_Mo_7_O_24_ (0.01 μM), Fe‐EDTA (200 μM). The Cd stress with or without MeJA was imposed after 4 days (10 days after sowing, DAS) by adding respective concentrations of Cd (10 and 20 µM CdCl_2_·H_2_O; Sigma-Aldrich) and MeJA (2.5 and 5 µM; Sigma-Aldrich) into the nutrient solution. The hydroponic experiment was established maintaining a completely randomized design with three replications. A single tank with 14 plants was treated as a single replicate and therefore, 21 tanks (7 × 3; treatments × replications) were maintained. Continuous aeration was provided in the hydroponic solutions by air pumps and the solutions were replaced in 6-day intervals. The duration of Cd exposure along with MeJA application was 18 days and the total experimental period was 4 weeks. The hydroponic system was maintained in a controlled growth room. The plants received artificial photosynthetic photon flux density (PPFD) of approximately 300 μmol m^−2^ s^−1^ provided by 60 W LED tubes maintaining 12 h photoperiod and 25/20 °C (day/night) temperature.

### Morphological measurements

The 4-week-old water spinach plants of seven growth conditions were harvested for the collection of morphological traits. A total of six plants from each treatment were randomly selected (two from each tank) for morphological data recording. The root and shoot length (RL and SL) were taken in cm with a 1-m ruler. The fresh weight (mg) of different plant parts such as root (RFW), stem (SFW), leaf (LFW) and total fresh weight (TFW = RFW + SFW + LDW) were measured by a digital weighing balance. Similarly, the dry weight of root (RDW), stem (SDW) and leaf (LDW) were acquired by oven drying the respective plant samples at 70 °C for 72 h and calculated the total dry weight (TDW = RDW + SDW + LDW). The root-shoot ratio (RSR) was calculated as RL over SL. The number of leaves per plant was counted and the leaf area (LA; cm^2^ plant^−1^) was determined using a leaf area meter (LI-3100C, LI-COR Environmental, Lincoln, USA). The specific leaf area (SLA) was estimated as leaf area to leaf dry weight.

### Gas exchange parameters and leaf greenness determination

The gas exchange measurements and leaf greenness were also made on the 4 weeks old plants. Well-developed broad leaves of three plants were selected for taking gas exchange attributes using a portable photosynthetic system (LC*i*-SD Photosynthetic system, ADC Bio Scientific Ltd., Hertfordshire, UK). The net photosynthesis (*A*, µmol CO_2_ m^−2^ s^−1^), stomatal conductance (*g*_*s*_, mol m^−2^ s^−1^), and rate of transpiration (*E*, mmol m^−2^ s^−1^) were measured at 200 µmol m^−2^ s^−1^ PPFD with ambient air temperature (25 °C) and CO_2_ levels (400 ppm). The leaf greenness or the index of leaf chlorophyll was estimated by a handheld SPAD meter (SPAD-502, Konica Minolta, Osaka, Japan). The SPAD values were logged at the three positions of a single leaf (base, middle, and top) and the mean value was considered as a single replicate and used for data analysis.

### Pigment contents analysis

The chlorophyll a (Chl *a*), chlorophyll b (Chl *b*), total chlorophyll (Total Chl) and total carotenoids contents (Total Car) were measured following the protocol described by Lichtenthaler^50^. Approximately 50 mg fresh leaf sample was dipped in 10 mL of 80% acetone and left in dark for 7 days for complete extraction of pigments. A UV–Vis spectrophotometer (DR6000, Hach, Dusseldorf, Germany) was used to obtain absorbances at 470, 646.8 and 663.2 nm wavelengths and calculated the pigment contents (mg g^−1^ FW) using the proposed formula of Lichtenthaler^50^.

### Determination of Lipid peroxidation (MDA content) in root and leaf

Root and leaf lipid peroxidation were analyzed as malondialdehyde (MDA) contents following TBARS assay^51^ with minor modifications^52^. The absorbance of the supernatants was measured at 532 and 600 nm and calculated the MDA content (nmol g^−1^ FW) using the subtracting values from 532 to 600 nm and the extinction coefficient of 155 mM^−1^ cm^−1^.

### H_2_O_2_ determination in root and leaf

The hydrogen peroxide (H_2_O_2_) contents in root and leaves were determined corresponding to Alexieva et al.^53^. The 0.1% TCA was used to homogenize 0.1 g fresh sample, and the homogenate was centrifuged at 12,000 rpm for 15 min at 4 °C. A reaction mixture of 1.5 mL was prepared by mixing 10 mM KH_2_PO_4_ buffer (pH 7.0), 1 M KI and plant extract; the blank contained 0.1% TCA only. The reaction was facilitated in darkness for an hour, and the optical density was measured at 390 nm. A standard curve of H_2_O_2_ was constructed using known concentrations and the H_2_O_2_ content (µmol g^−1^ FW) was calculated from the standard curve.

### Proline contents in root and leaf

The root and leaf proline contents were estimated following the protocol of Carillo et al.^54^ with minor modifications^52^. Approximately 50 mg fresh samples were blended in 70% ethanol and centrifuged the homogenate for 5 min at 12,000 rpm. A reaction mixture containing plant extract, 1% Ninhydrin (*w/v*) in 60% acetic acid (*v/v*) and 20% ethanol (*v/v*) was prepared and incubated at 95 °C for 90 min in a water bath to initiate the reaction. The reaction was stopped by cooling the tubes at room temperature and then the absorbance was recorded at 520 nm using the spectrophotometer. Quantification of proline content (µg g^−1^ FW) from the absorbance values was obtained from a standard curve of l-proline (Sigma-Aldrich).

### Enzymatic antioxidants and total antioxidant capacity (TAC) analysis

The extraction process outlined by Elavarthi and Martin^55^ was followed to extract the root and leaf samples for the activities of superoxide dismutase (SOD, 1.15.1.1), catalase (CAT, EC1.11.1.6) and guaiacol peroxidase (POD, EC1.11.1.7) enzymes. Approximately 100 mg of leaf samples were pulverized with KH_2_PO_4_ buffer (pH 7.0) in a pre-cooled mortar and pestle, and the mixture was centrifuged at 12,000 rpm for 20 min at 4 °C.

The SOD activity was assayed by determining its capability to impair the photochemical reduction of nitro blue tetrazolium (NBT) as explained by Beauchamp and Fridovich^56^ with slight modifications. The 3 mL assay consisted of KH_2_PO_4_buffer (50 mM, pH 7.8), NBT (75 µM), l-methionine (13 mM), EDTA (0.1 mM), riboflavin (2 µM) and 50 µL of plant extract. Riboflavin was added last, and tubes were placed 30 cm below a light source (15 W fluorescent lamps) to initiate the reaction with gentle shaking and allowed for 15 min. The reaction mixture except plant extract was used as control (irradiated) and blank (non-irradiated) and ran in parallel. The control tubes lacking enzymes developed maximum bluish color whereas the blank sample did not develop color. The absorbance readings of all samples were taken at 560 nm using spectrophotometer. SOD activities of leaf and root were expressed as unit min^−1^ g^−1^ FW where, one unit of SOD was termed as the quantity of enzyme that inhibits NBT photoreduction by 50% per minute.

For CAT, an assay of 3 mL was prepared with a combination of 100 µL supernatants, 10 mM H_2_O_2_ and 50 mM KH_2_PO_4_ buffer (pH 7.0). The activity of CAT was acquired by observing the decrease in the rate of absorbances in 30 s intervals (total period of 90 s) at 240 nm wavelength by a *UV–VIS* spectrophotometer (DR6000, Hach, Dusseldorf, Germany). The CAT activity (mmol min^−1^ g^−1^ FW) was calculated using the extinction coefficient of H_2_O_2_ (40 mM^−1^ cm^−1^)^57^. For POD determination, a 3 mL assay was made with 50 µL sample extract, 2 mM H_2_O_2_, 5 mM Guaiacol, and 50 mM KH_2_PO_4_ buffer (pH 7.0). Plant samples were mixed later to initiate the reaction and an increase in absorbances in 30 s interval (90 s total duration) was recorded at 470 nm wavelength using the spectrophotometer. The activity of POD (mmol min^−1^ g^−1^ FW) was obtained using the extinction coefficient of Tetraguaiacol (26 mM^−1^ cm^−1^)^58^. Total antioxidant activity (TAC) of the root and leaf of water spinach plants was obtained following the phosphomolybdate method as referred by Prieto et al.^59^ with minor modifications^52^. The absorbance reading was taken at 695 nm, and the TAC (mg equivalent AA g^−1^ FW) was determined from a reference standard of ascorbic acid (AA).

### Cd determination in root, stem and leaf

The harvested plants of water spinach were partitioned into roots, stems and leaves and then oven-dried at 70 °C until receiving constant weight. About 0.5 g of respective dried samples were ground and digested with 20 mL nitric acid and 10 mL perchloric acid. The digest was kept on a hot plate at 160–220 °C until the solution became colorless. The absorbance reading was taken at 228.8 nm wavelength for Cd determination with an atomic absorption spectrophotometer (AA-7000, Shimadzu, Kyoto, Japan).

### Statistical data analysis

The statistical analysis of the recorded data was performed by the open-source programming of R^60^ *v. 4.0.5* accessed in April 2021. Analysis of Variance (ANOVA) was executed considering the treatment means and the multiple comparisons of treatment means were performed following the Tukey HSD test at a significance level of *p* < 0.05. The stress tolerance index (STI) of the measured traits was computed as (stress values/control values) × 100. The normalized STI values of 40 traits were considered to construct the hierarchical clustering heatmap using the *ComplexHeatmap* package of the R program. The principal component analysis (PCA) biplot and correlation matrix were created following the *fviz_pca* and *corrplot* functions of the R statistical software.

## Results

### Morphological traits

**Fig. 1:**
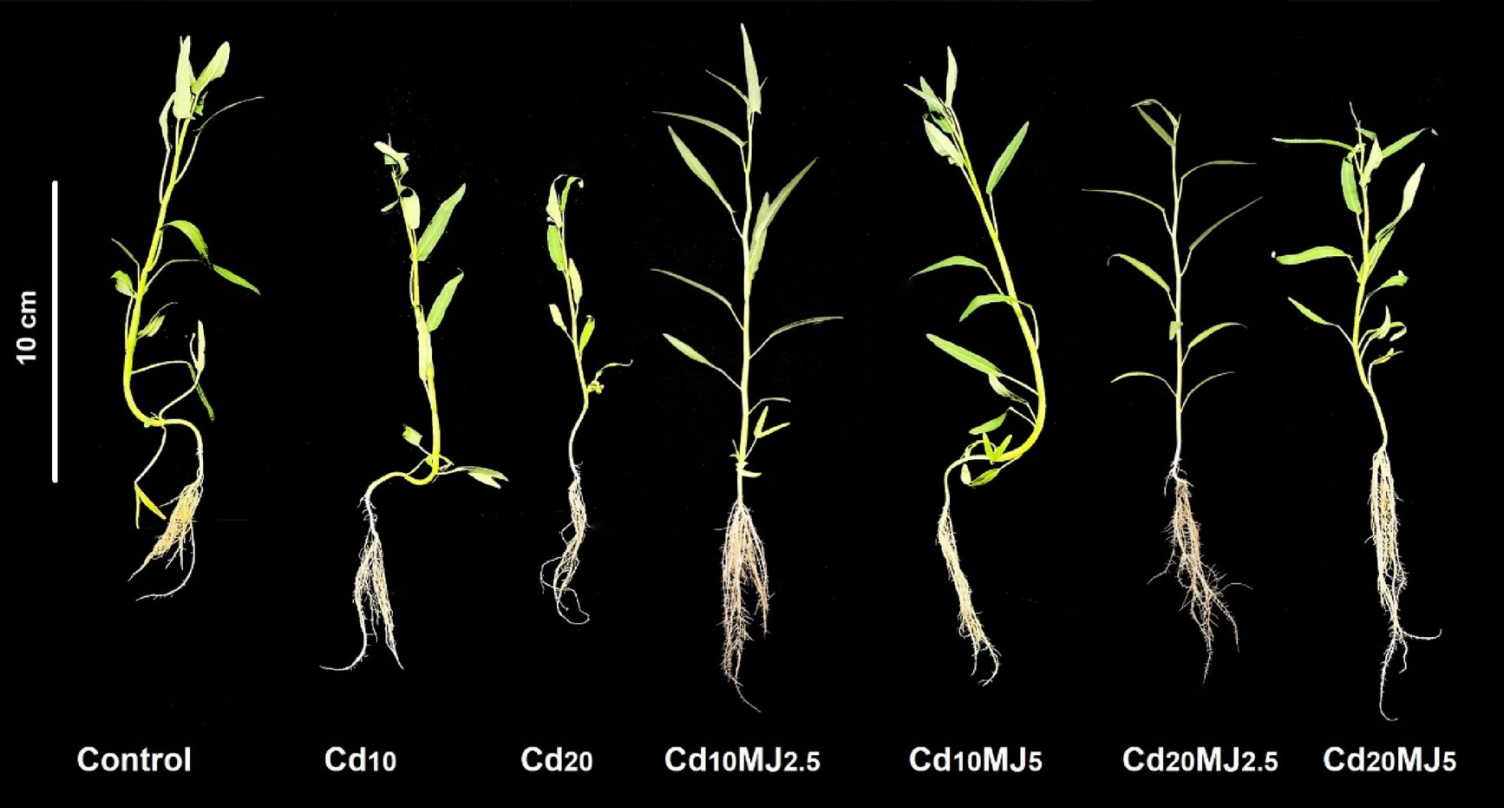
28-Day-old water spinach plants grown in different growth conditions (Control, Cd_10_ = 10 µM Cd, Cd_20_ = 20 µM Cd, Cd_10_MJ_2.5_ = 10 µM Cd + 2.5 µM MeJA, Cd_10_MJ_5_ = 10 µM Cd + 5 µM MeJA, Cd_20_MJ_2.5_ = 20 µM Cd + 2.5 µM MeJA, Cd_20_MJ_5_ = 20 µM Cd + 5 µM MeJA).

Plant growth attributes including root and shoot length, root shoot ratio, leaf number, leaf area, specific leaf area and plant biomass were greatly altered due to Cd stress with or without exogenous MeJA supplementation (Tables 1 and 2, Fig. 1). The longest root length (RL) ranged from 15.7 cm in control to 22.6 cm in Cd_20_MeJA_2.5_ (Table 1, Fig. 1). In general, the root length was longer in Cd treated plants compared to control and the longest root length increased with the elevation of Cd levels. The shoot length (SL) in Cd_10_ and Cd_20_ treatments decreased by 26% and 32% compared to the control. MeJA supplementation with 2.5 µM increased SL by 25% and 32% while 5 µM increased by 15% and 18% compared to Cd_10_ and Cd_20_ treatments, respectively (Table 1, Fig. 1). Root-shoot ratio (RSR) varied significantly among treatments ranging from 0.35 to 0.71 (Table 1). Among MeJA treatments, the maximum RSR was computed in Cd_20_MJ_2.5_ (0.63) while the minimum in Cd_10_MJ_5_ (0.42). The number of leaves per plant was reduced by 22% and 27% in Cd_10_ and Cd_20_ treatments, respectively compared to control while the variations in leaf number between control and MeJA treated plants were insignificant. Compared to control, LA decreased by 35% and 52% in Cd_10_ and Cd_20_ treatments, respectively whereas these reductions were significantly lesser in Cd_10_MJ_5_ (8%), Cd_10_MJ_2.5_ (26%), Cd_20_MJ_5_ (32%) and Cd_20_MJ_2.5_ (38%) (Table 1). The plant fresh and dry mass gradually decreased with the increase of Cd levels and varied significantly among treatments the maximum fresh and dry weights of all plant parts were recorded in Cd_10_MJ_5_ treatment (Table 2). The TDW declined by 56% and 62% in Cd_10_ and Cd_20_ treatments, respectively compared to control (Table 2). The exogenous application of MeJA alleviated the Cd-suppressed dry mass production in water spinach plant with an increase of 28%, 66%, 32% and 38% TDW in Cd_10_MJ_2.5_, Cd_10_MJ_5_, Cd_20_MJ_2.5_, Cd_20_MJ_5_, respectively.

**Table 1:** Morphological traits of water spinach plants grown in different growth conditions.

Treatments	Longest RL (cm plant^−1^)	SL (cm plant^−1^)	RSR	Leaf number (plant^−1^)	LA (cm^2^ plant^−1^)	SLA (cm^2^ g^−1^)
Control	15.7 ± 1.2 b	45.8 ± 3.6 a	0.35 ± 0.03 c	11.3 ± 0.8 a	62.3 ± 1.6 a	0.40 ± 0.009 b
Cd_10_	18.8 ± 1.1 ab	33.8 ± 2.5 cd	0.57 ± 0.08 a–c	8.8 ± 0.3 b	40.7 ± 2.8 b	0.49 ± 0.010 a
Cd_20_	21.7 ± 1.0 ab	31.3 ± 2.3 d	0.71 ± 0.07 a	8.3 ± 0.3 b	30.0 ± 2.4 c	0.41 ± 0.010 b
Cd_10_MJ_2.5_	22.5 ± 1.9 a	42.1 ± 0.7 a–c	0.54 ± 0.05 a–c	11.7 ± 0.3 a	46.0 ± 0.9 b	0.4 ± 0.023 ab
Cd_10_MJ_5_	18.7 ± 1.6 ab	44.5 ± 1.3 ab	0.42 ± 0.04 bc	11.3 ± 0.5 a	57.1 ± 2.7 a	0.42 ± 0.025 ab
Cd_20_MJ_2.5_	22.6 ± 1.5 a	36.0 ± 1.0 b–d	0.63 ± 0.05 ab	9.8 ± 0.3 ab	38.9 ± 2.0 bc	0.39 ± 0.015 b
Cd_20_MJ_5_	20.1 ± 1.2 ab	36.8 ± 1.0 a–d	0.55 ± 0.04 ac	10.0 ± 0.4 ab	42.4 ± 1.1 b	0.42 ± 0.015 ab

Treatment means with different letters in a column denote significant differences at 5% levels of probability. Values are treatment mean ± SEM (n = 6). Treatment description: Cd_10_ = 10 µM Cd, Cd_20_ = 20 µM Cd, Cd_10_MJ_2.5_ = 10 µM Cd + 2.5 µM MeJA, Cd_10_MJ_5_ = 10 µM Cd + 5 µM MeJA, Cd_20_MJ_2.5_ = 20 µM Cd + 2.5 µM MeJA, Cd_20_MJ_5_ = 20 µM Cd + 5 µM MeJA. Traits description: *RL* root length, *SL* shoot length, *RSR* root-shoot ratio, *LA* leaf area, *SLA* specific leaf area.

**Table 2:** Fresh and dry mass of different parts of water spinach plants grown in different growth conditions.

Treatments	RFW (mg plant^−1^)	SFW (mg plant^−1^)	LFW (mg plant^−1^)	TFW (mg plant^−1^)	RDW (mg plant^−1^)	SDW (mg plant^−1^)	LDW (mg plant^−1^)	TDW (mg plant^−1^)
Control	646 ± 47 b	4626 ± 267 a	1334 ± 35 a	6605 ± 294 a	108 ± 3 a	302 ± 11 a	163 ± 4 a	566 ± 10 a
Cd_10_	732 ± 39 ab	2615 ± 119 c	837 ± 52 c	4183 ± 98 d	46 ± 4 de	122 ± 3 cd	84 ± 5 cd	252 ± 5 de
Cd_20_	594 ± 33 b	2062 ± 98 c	677 ± 37 c	3333 ± 110 e	39 ± 2 e	103 ± 5 d	74 ± 5 d	216 ± 8 e
Cd_10_MJ_2.5_	755.8 ± 45 ab	2490.8 ± 64 c	1212.4 ± 57 ab	5001 ± 280 bc	50 ± 3 cde	126 ± 4 cd	111 ± 7 cd	321 ± 17 c
Cd_10_MJ_5_	892 ± 37 a	3466 ± 73 b	1225 ± 30 ab	5582 ± 95 b	65 ± 4 b	216 ± 6 cd	136 ± 6 b	416 ± 7 b
Cd_20_MJ_2.5_	736.7 ± 21 ab	2339.0 ± 25 c	1084.0 ± 49 b	4160 ± 72 d	58 ± 2 bcd	126 ± 2 cd	100 ± 6 bc	283 ± 7 cd
Cd_20_MJ_5_	822 ± 31 a	2478 ± 72 c	1105 ± 37 b	4404 ± 120 cd	61 ± 3 bc	134 ± 5 c	102 ± 4 bc	296 ± 9 c

Treatment means with different letters in a column denote significant differences at 5% levels of probability. Values are treatment mean ± SEM (n = 6). Treatment description: Cd_10_ = 10 µM Cd, Cd_20_ = 20 µM Cd, Cd_10_MJ_2.5_ = 10 µM Cd + 2.5 µM MeJA, Cd_10_MJ_5_ = 10 µM Cd + 5 µM MeJA, Cd_20_MJ_2.5_ = 20 µM Cd + 2.5 µM MeJA, Cd_20_MJ_5_ = 20 µM Cd + 5 µM MeJA. Traits description: *RFW* fresh weight of root, *SFW* fresh weight of stem, *LFW* leaf fresh weight, *TFW* total fresh weight, *RDW* dry weight of root, *SDW* stem dry weight, *LDW* leaf dry weight, *TDW* total dry weight.

### Gas exchange parameters and leaf greenness (SPAD)

**Fig. 2:**
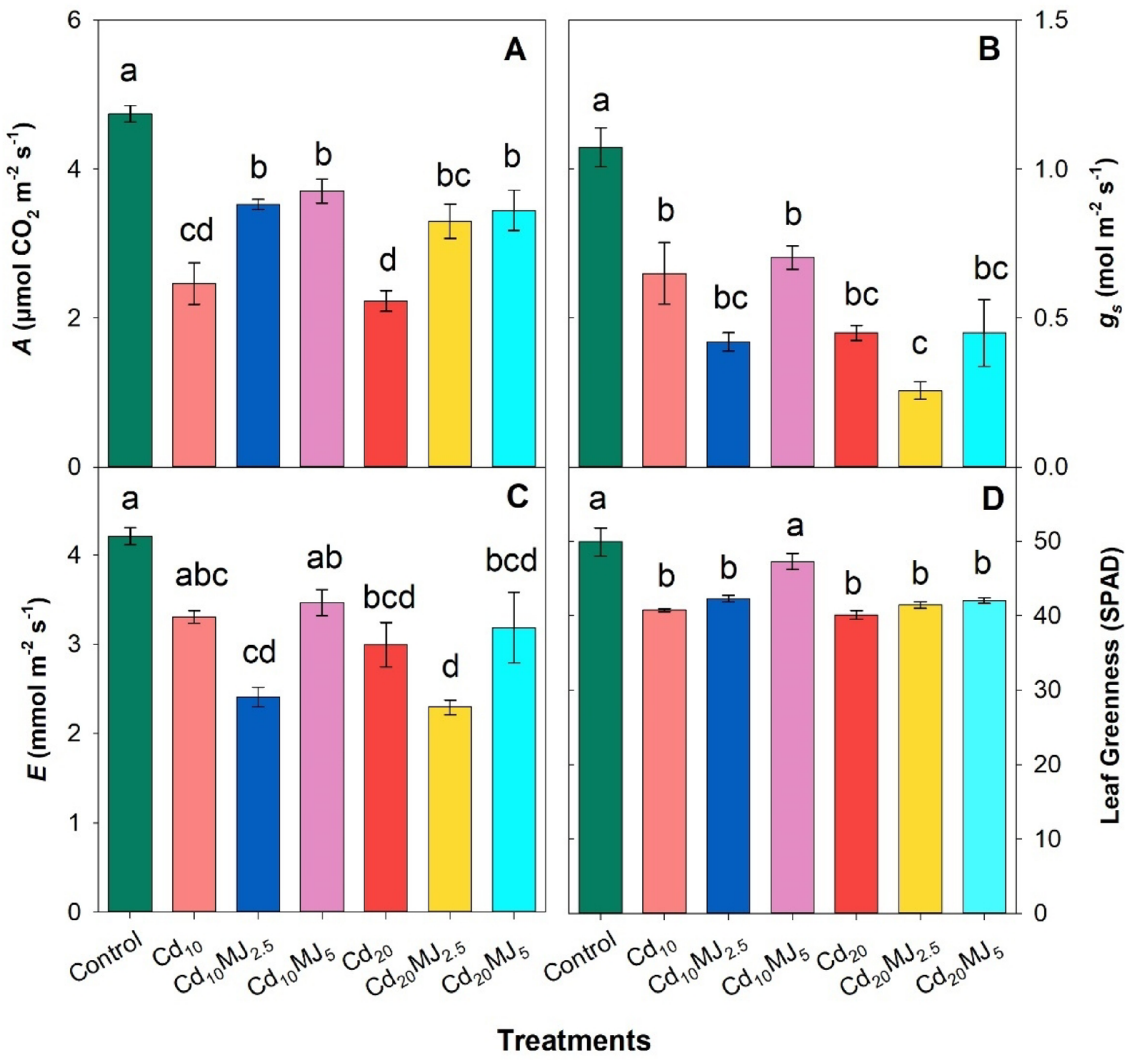
(**A**) Photosynthesis rate (*A*), (**B**) stomatal conductance (*g*_*s*_), (**C**) transpiration rate (*E*), and (**D**) leaf greenness (SPAD value) of 28 days old hydroponically grown water spinach plants at different growth conditions. The vertical bars represent SEM (n = 3). Treatment means with different letters imply significant at 5% levels of probability. Treatment description: Cd_10_ = 10 µM Cd, Cd_20_ = 20 µM Cd, Cd_10_MJ_2.5_ = 10 µM Cd + 2.5 µM MeJA, Cd_10_MJ_5_ = 10 µM Cd + 5 µM MeJA, Cd_20_MJ_2.5_ = 20 µM Cd + 2.5 µM MeJA, Cd_20_MJ_5_ = 20 µM Cd + 5 µM MeJA.

Gas exchange parameters in water spinach leaves were remarkably altered by both levels of Cd exposure (Fig. 2A–C). The *A* was reduced by 48% and 53% in Cd_10_ and Cd_20_ treated leaves, respectively compared to control. MeJA supplementation significantly improved the *A* displaying least reductions in Cd_10_MJ_2.5_ (26%), Cd_10_MJ_5_ (22%), Cd_20_MJ_2.5_ (30%) and Cd_20_MJ_5_ (27%) treatments in relation to control (Fig. 2A). The *g*_*s*_ substantially varied among treatments showing a peak at control followed by Cd_10_MJ_5_ (8% higher than Cd_10_) while lowest *g*_*s*_ was recorded in Cd_20_MJ_2.5_ (Fig. 2B). The variations in *E* between control and all Cd_10_ treatments were insignificant except Cd_10_MJ_2.5_ whereas the Cd_20_ treatments showed significant differences with control (Fig. 2C). A slight increase in *E* (5% and 6%) was observed in Cd_10_MJ_5_ and Cd_20_MJ_5_ compared to Cd_10_ and Cd_20_ treatments, respectively. Cd stress significantly declined leaf greenness (SPAD value) in relation to the Cd-free plants. The treatment Cd_10_MJ_5_ showed a considerable increase in leaf greenness by 16% compared to the 10 μM Cd stress alone (Fig. 2D). Conversely, the other MeJA treatments under Cd stresses appeared to have insignificant alterations among them (Fig. 2D).

### Leaf pigments

**Fig. 3:**
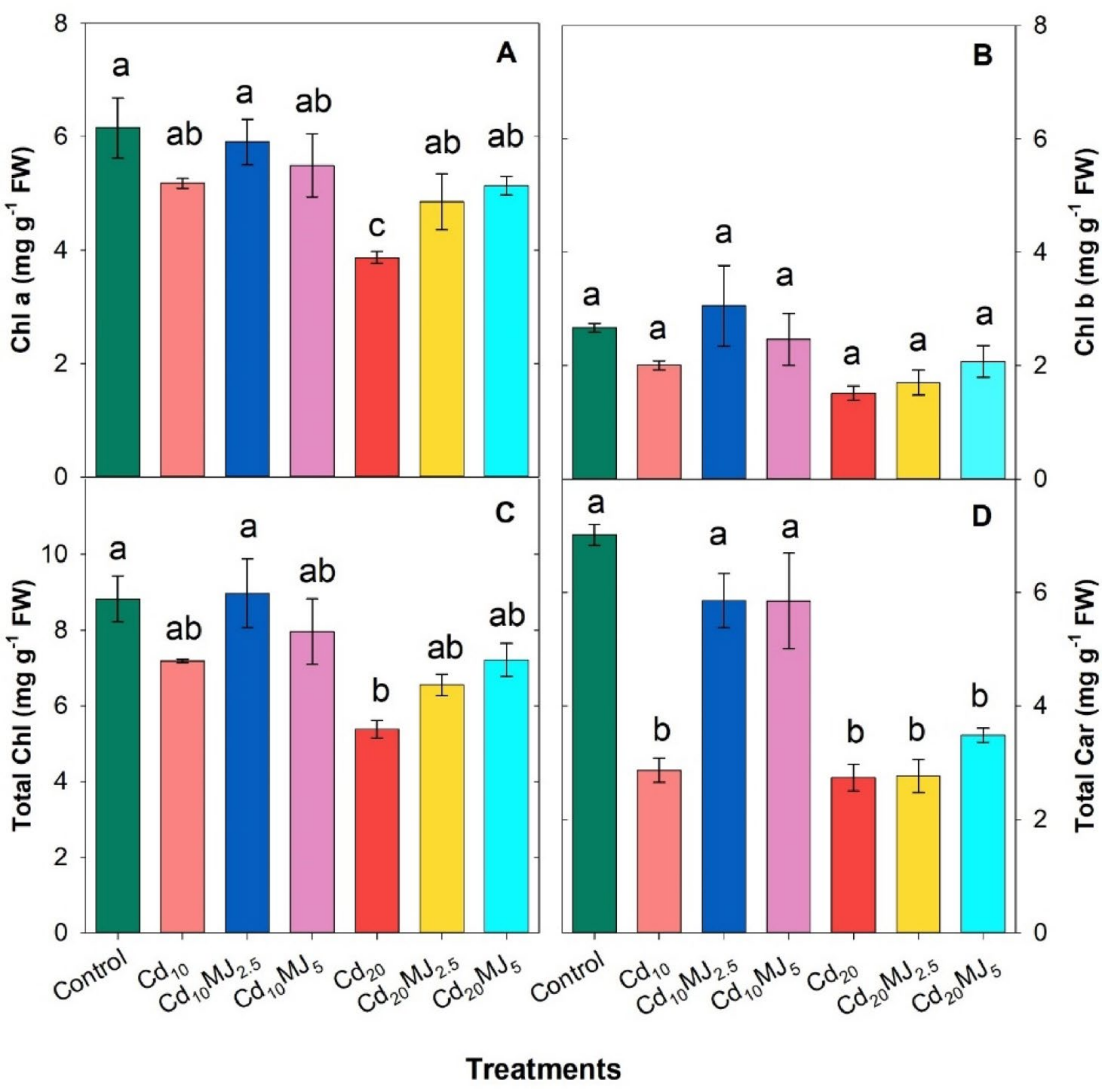
Chlorophyll a (Chl *a*), Chlorophyll b (Chl *b*), Total Chlorophyll (Total Chl) and Total carotenoids (Total Car) contents in the leaf of 28 days old hydroponically grown water spinach plants at different growth conditions. The vertical bars represent SEM (n = 3). Treatment means with different letters imply significant at 5% levels of probability. Treatment description: Cd_10_ = 10 µM Cd; Cd_20_ = 20 µM Cd; Cd_10_MJ_2.5_ = 10 µM Cd + 2.5 µM MeJA, Cd_10_MJ_5_ = 10 µM Cd + 5 µM MeJA; Cd_20_MJ_2.5_ = 20 µM Cd + 2.5 µM MeJA, Cd_20_MJ_5_ = 20 µM Cd + 5 µM MeJA.

Cd exposure considerably lowered the leaf Chl *a*, Chl *b*, Total Chl and Total Car contents of water spinach plant in all treatments except Cd_10_MJ_2_._5_ while MeJA treatment fairly recovered leaf pigments contents upon Cd stresses (Fig. 3). There were no significant variations among the treatments concerning Chl *b* content. In 20 μM Cd level, the supplementation of 5 μM MeJA significantly increased leaf Chl *a* (32.75%) whereas the rise of Chl *b*, Total Chl and Total Car contents were not statistically differed in contrast to Cd_20_ treated leaves (Fig. 3A–D). Besides, Total Car content was almost doubled in Cd_10_MJ_2.5_ and Cd_10_MJ_5_ compared to Cd_10_ treatment while no significant variation was observed among all Cd_20_-treated plants (Fig. 3D).

### H_2_O_2_ and MDA contents in root and leaf

**Fig. 4:**
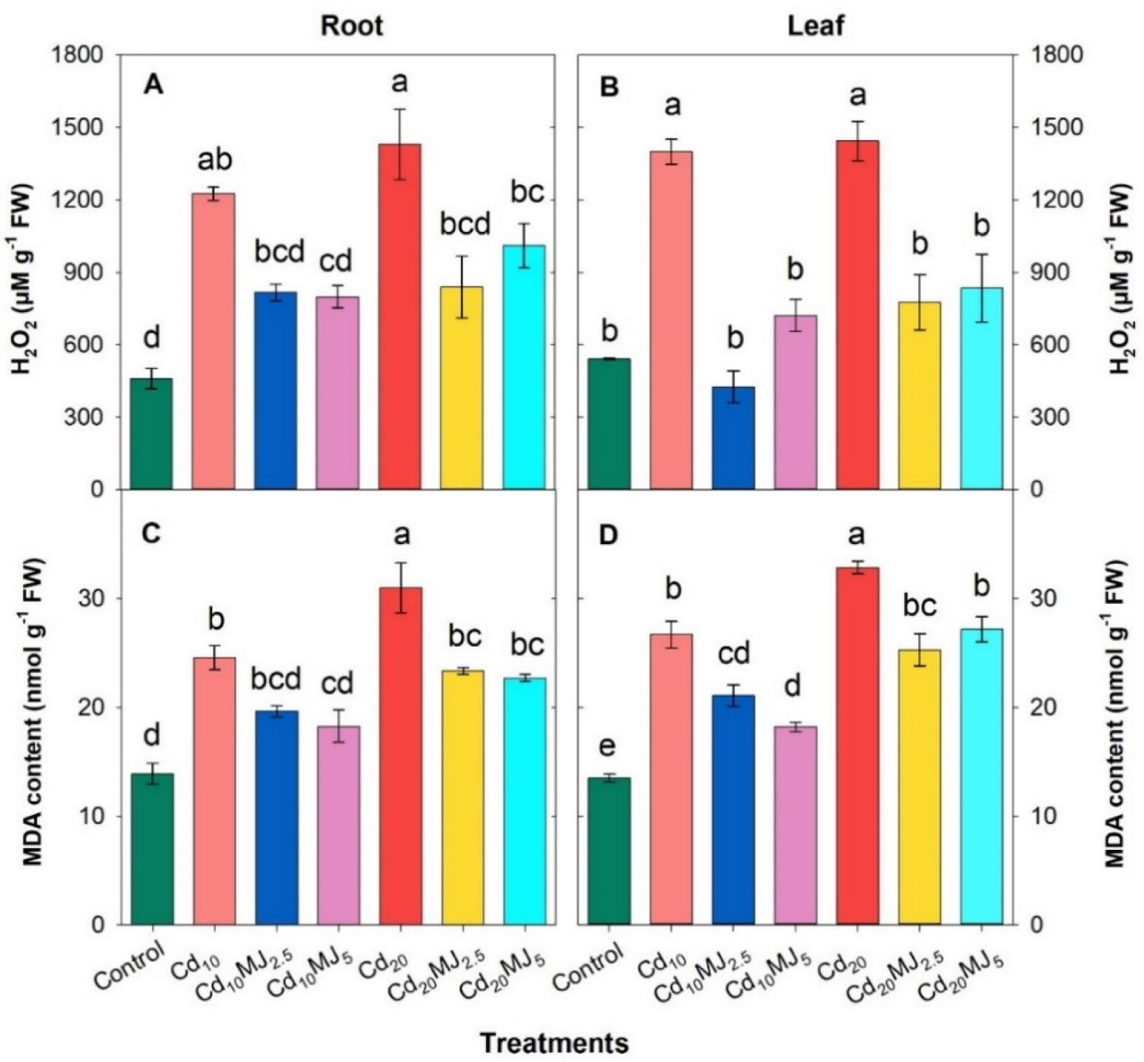
Hydrogen peroxide (H_2_O_2_) and lipid peroxidation (MDA content) in root and leaf of 28 days old hydroponically grown water spinach plants at different growth conditions. The vertical bars represent SEM (n = 3). Treatment means with different letters imply significant at 5% levels of probability. Treatment description: Cd_10_ = 10 µM Cd, Cd_20_ = 20 µM Cd, Cd_10_MJ_2.5_ = 10 µM Cd + 2.5 µM MeJA, Cd_10_MJ_5_ = 10 µM Cd + 5 µM MeJA, Cd_20_MJ_2.5_ = 20 µM Cd + 2.5 µM MeJA, Cd_20_MJ_5_ = 20 µM Cd + 5 µM MeJA.

Cadmium stress substantially induced lipid peroxidation by rising MDA and H_2_O_2_ contents in roots and leaves of water spinach plants (Fig. 4A–D). MeJA application significantly suppressed Cd-induced oxidative damage through the lower accumulation of MDA and H_2_O_2_ in the root and leaf (Fig. 4). The plants treated with 5 μM MeJA showed significant reduction in root MDA (26% and 27%) and H_2_O_2_ (35% and 29%) contents in comparison to Cd_10_ and Cd_20_-treated plants, respectively (Fig. 4A,C). Similarly, the leaf MDA contents were reduced by 27%, 32%, 23% and 17% and H_2_O_2_ contents by 228%, 49%, 46% and 42% in Cd_10_MJ_2.5_, Cd_10_MJ_5_, Cd_20_MJ_2.5_ and Cd_20_MJ_5_ treatments, respectively while comparing with their individual Cd treatment (Fig. 4B,D).

### Total antioxidant activity (TAC) and proline content in root and leaf

**Fig. 5:**
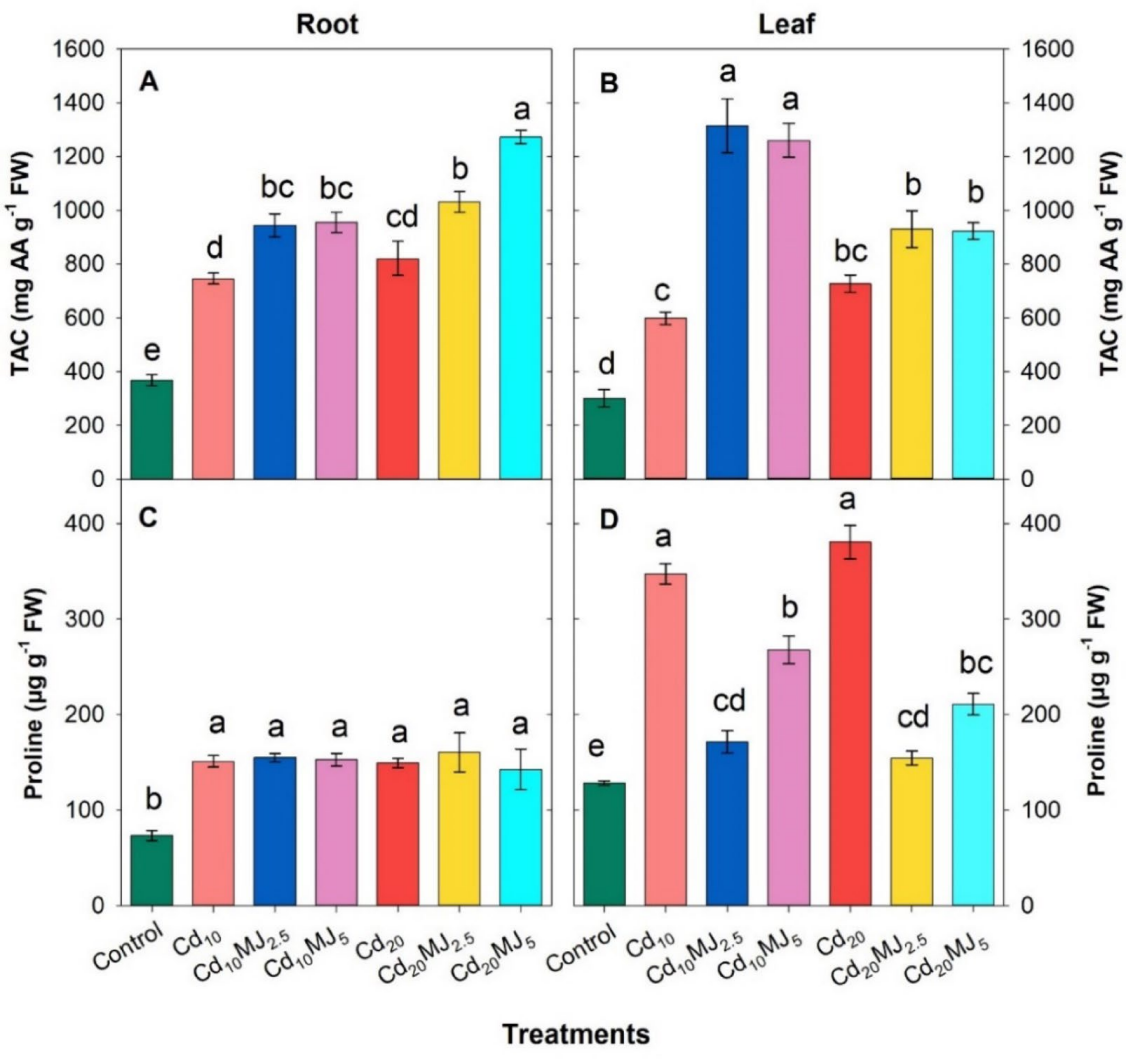
Total Antioxidant Capacity (TAC) and Proline content in root and leaf of 28 days old hydroponically grown water spinach plants at different growth conditions. The vertical bars represent SEM (n = 3). Treatment means with different letters imply significant at 5% levels of probability. Treatment description: Cd_10_ = 10 µM Cd; Cd_20_ = 20 µM Cd; Cd_10_MJ_2.5_ = 10 µM Cd + 2.5 µM MeJA, Cd_10_MJ_5_ = 10 µM Cd + 5 µM MeJA; Cd_20_MJ_2.5_ = 20 µM Cd + 2.5 µM MeJA, Cd_20_MJ_5_ = 20 µM Cd + 5 µM MeJA.

The water spinach plant exhibited a significant surge in root and leaf total antioxidant capacity (TAC) and proline content in all Cd treatments compared to control (Fig. 5A–D). Compared to Cd_10_, root TAC rose gradually by 27% and 28% in Cd_10_MJ_2.5_ and Cd_10_MJ_5_, respectively while root TAC in Cd_20_MJ_5_ increased by 55% relative to Cd_20_ (Fig. 5A). Similar trend was observed in the case of leaf TAC where MeJA supplementation doubled (120% and 111%) the leaf TAC at 10 μM Cd levels (Fig. 5B). Results revealed that the augmentation of root proline under Cd with or without MeJA application was almost doubled over control whereas the six Cd treatments did not show any significant variations in root proline content among themselves (Fig. 5C). Leaf proline content uplifted progressively by 170% and 196% in Cd_10_ and Cd_20_ treatments compared to control, respectively (Fig. 5D). Nevertheless, MeJA supplementation lowered leaf proline contents compared to Cd-alone treatments. MeJA in both levels decreased leaf proline contents by 51% and 23% compared to Cd_10_ and by 59% and 45% compared to Cd_20_, respectively (Fig. 5D).

### Antioxidative enzymes activities

**Fig. 6:**
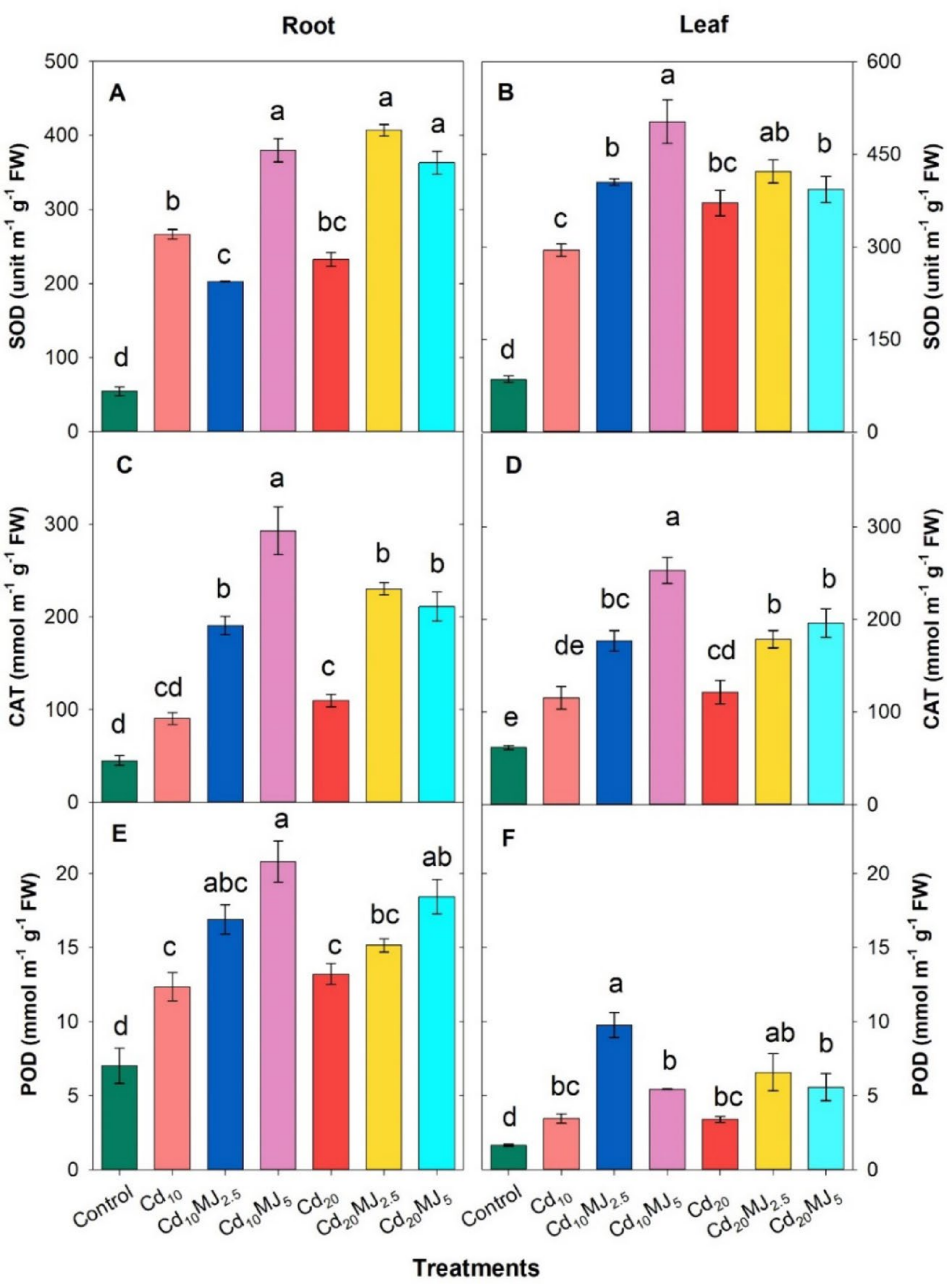
Activities of superoxide dismutase (SOD), catalase (CAT), and peroxidase (POD) in root and leaf of 28 days old hydroponically grown water spinach plants at different growth conditions. The vertical bars represent SEM (n = 3). Treatment means with different letters imply significant at 5% levels of probability. Treatment description: Cd_10_ = 10 µM Cd, Cd_20_ = 20 µM Cd, Cd_10_MJ_2.5_ = 10 µM Cd + 2.5 µM MeJA, Cd_10_MJ_5_ = 10 µM Cd + 5 µM MeJA, Cd_20_MJ_2.5_ = 20 µM Cd + 2.5 µM MeJA, Cd_20_MJ_5_ = 20 µM Cd + 5 µM MeJA.

Cd exposure showed a remarkable raise in SOD, CAT and POD activities in the root and leaves of water spinach plant (Fig. 6). The root SOD, CAT, and POD activities in Cd_10_ treatment were significantly enhanced by 388%, 101% and 76% compared to control while in Cd_20_, the increments were 326%, 145% and 89% over control, respectively (Fig. 6A,C,E). Likewise, the leaf SOD, CAT, and POD activities were augmented by 245%, 87% and 111% in Cd_10_ and by 334%, 97% and 106% inCd_20_ compared to control, respectively (Fig. 6B,D,F). Moreover, further remarkable elevation of SOD, CAT and POD activities were obtained in the root and leaf of water spinach when treated with MeJA exogenously. The highest induction of SOD activity in the root was observed at Cd_20_MJ_2.5_ (75% higher than Cd_20_) and that of root CAT and POD were in Cd_10_MJ_5_ (224% and 69% higher than Cd_10_) (Fig. 6A,C,E). The SOD and CAT activities in the leaves were maximum in Cd_10_MJ_5_ (71% and 120% higher than Cd_10_) while the greater activity of leaf POD was found in Cd_10_MJ_2.5_ (183% greater than Cd_10_) (Fig. 6B,D,F).

### Cd contents in root, stem and leaf

**Fig. 7:**
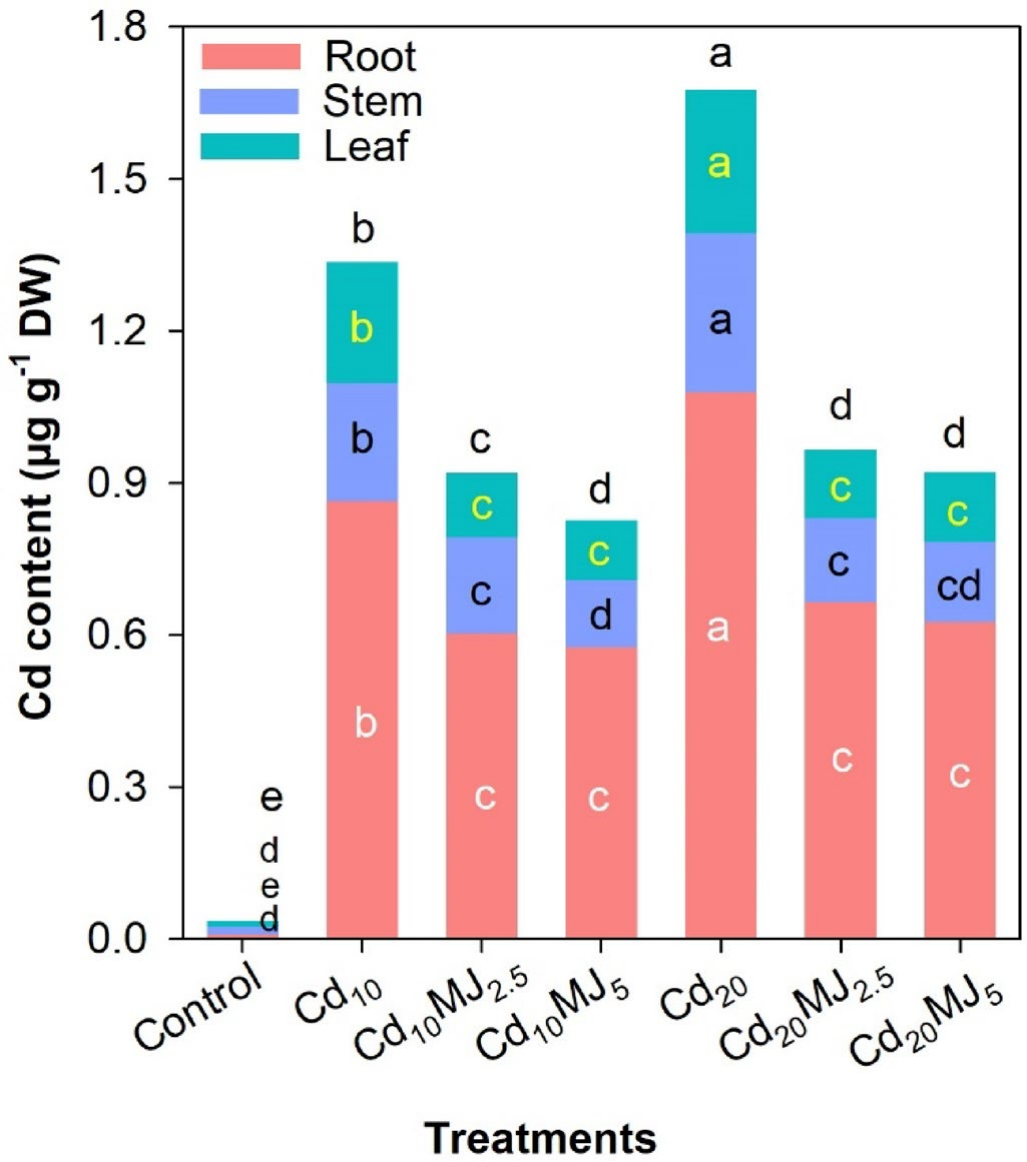
Cd contents (µg g^−1^ DW) in roots, stems and leaves of 28 days old water spinach plant grown in seven growth conditions. Treatment means with different letters in the middle portion of the respective colored bars indicate significant differences at 5% levels of probability. Treatment description: Cd_10_ = 10 µM Cd, Cd_20_ = 20 µM Cd, Cd_10_MJ_2.5_ = 10 µM Cd + 2.5 µM MeJA, Cd_10_MJ_5_ = 10 µM Cd + 5 µM MeJA, Cd_20_MJ_2.5_ = 20 µM Cd + 2.5 µM MeJA, Cd_20_MJ_5_ = 20 µM Cd + 5 µM MeJA.

Cd contents in roots, stems and leaves were greatly varied among the treatments (Fig. 7). The root Cd content was maximum in Cd_20_ followed by Cd_10_ while the lowest Cd content in root among MeJA treatments was recorded in Cd_10_MJ_5._ The stem and leaf Cd contents followed a similar trend as root (Fig. 7). The highest total Cd content was recorded in Cd_20_ (1.68 µg g^−1^ in total) followed by Cd_10_ (1.34 µg g^−1^ in total). Among MeJA treatments, the total Cd content in water spinach plant ranked as Cd_20_MJ_2.5_ > Cd_20_MJ_5_ > Cd_10_MJ_2.5_ > Cd_10_MJ_5_ (Fig. 7). The total Cd contents in Cd_10_MJ_2.5_, Cd_10_MJ_5_, Cd_20_MJ_2.5_ and Cd_20_MJ_5_ were decreased by 31%, 38%, 31% and 45%, respectively compared to the corresponding Cd-alone treatments. Cd contents in all treatments were found to be higher in the root in comparison to stem and leaf indicating a higher accumulation of Cd in the root than in other plant organs (Fig. 7). The greater translocation of Cd from root to shoot (stem + leaf) was observed in Cd_20_ (0.55) and Cd_10_ (0.54) while an extensive reduction of these translocations was carried out in Cd_10_MJ_2.5_ (0.52), Cd_10_MJ_5_ (0.44), Cd_20_MJ_2.5_ (0.45) and Cd_20_MJ_5_ (0.47). Results revealed that the supplementation of MeJA played a dual role in Cd tolerance—by lowering the uptake of Cd in the root and by reducing the translocation of Cd from root to shoot.

### Two-way hierarchical clustering heatmap

**Fig. 8:**
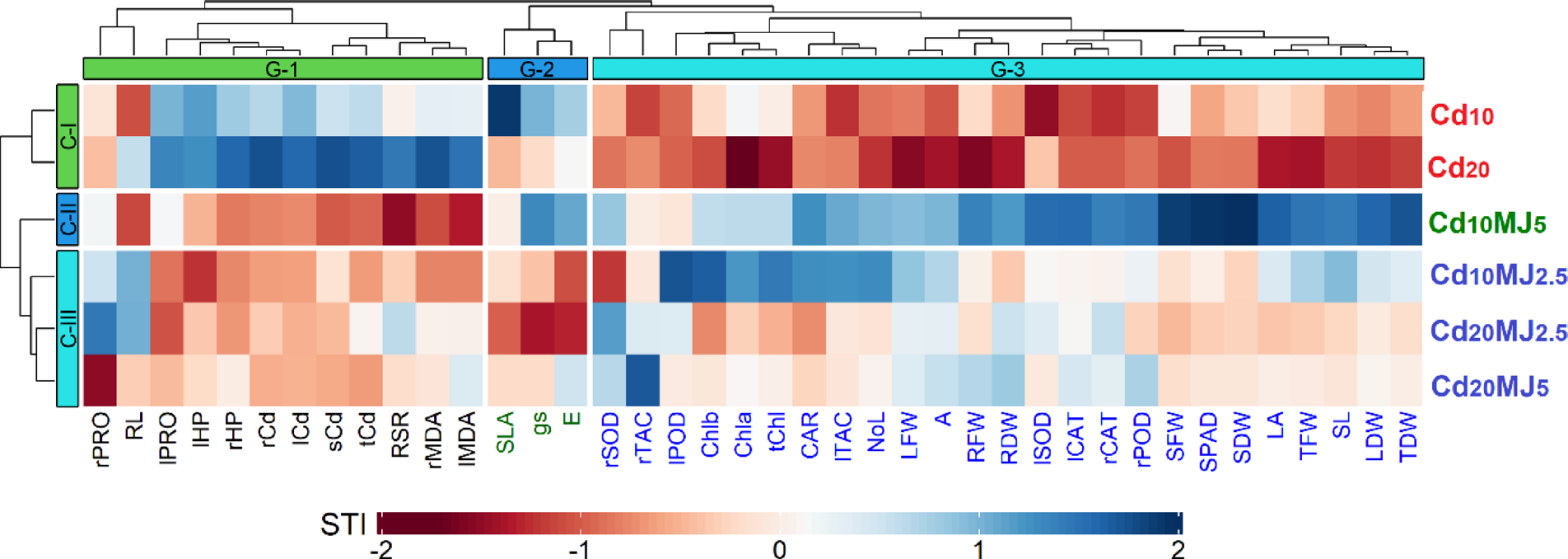
Two-way hierarchical clustering heatmap of 40 measured traits of water spinach plants grown in several Cd treatments. The standardized stress tolerance index (STI) values were used to create the heatmap. A single column represents a trait while a single row denotes a treatment. Colors correspond to a relative scale of − 2 to 2 where darker red and blue indicate higher STI and lower STI scores, respectively. Both the traits and treatments were clustered into three groups. Traits description: *r, s, l and t* root, stem, leaf and total, respectively, *PRO* Proline, *HP* H_2_O_2_, *Cd* Cadmium, *RL* Root length, *RSR* Root-shoot ratio, *MDA* Malonaldehyde, *SLA* Specific leaf area, *g*_*s*_ stomatal conductance, *E* Transpiration rate, *SOD* Superoxide dismutase, *TAC* Total antioxidant capacity, *POD* Peroxidase, *Chl* Chlorophyll, *CAR* Total carotenoids, *NoL* Number of leaf, *LFW* Leaf fresh weight, *A* Rate of photosynthesis, *RFW* Root fresh weight, *RDW* Root dry weight, *CAT* Catalase, *SFW* Stem fresh weight, *SDW* Stem dry weight, *LA* Leaf area, *TFW* Total fresh weight, *SL* Shoot length, *LDW* Leaf dry weight, *TDW* Total dry weight.

The degree of cadmium stress tolerance in water spinach is entirely explained by the two-way hierarchical clustering heatmap highlighting the STI values of the 40 measured traits (Fig. 8). The seven treatments were categorized (row-wise) into three clusters. The first cluster (C-I) consisted of Cd_10_ and Cd_20_ treatments, C-II with Cd_10_MJ_5_ and C-III with the other three treatments. On the other hand, the studied traits were also classified (column-wise) into three groups where Group-1 (G-1), Group-2 (G-2) and Group-3 (G-3) comprised of 12, 3 and 25 closely related traits, respectively. Generally, higher STI scores denote greater Cd tolerance in the case of the traits (G-1) those values were decreased under stress compared to the control. However, G-2 and G-3 traits followed the reverse pattern as the values of these traits were increased due to Cd stress in comparison to control. Considering this, the treatment Cd_10_MJ_5_ mostly reflected by darker blue in G-2 and G-3 traits and by darker red within G-1 traits exhibiting higher and lower STI scores, respectively. This explains a greater extent of Cd tolerance (least negative effects of Cd) in Cd_10_MJ_5_ compared to the other five treatments. Based on the STI scores extracted from the figure, the Cd tolerance among the treatments can be ranked as Cd_10_MJ_5_ > Cd_10_MJ_2_._5_ > Cd_20_MJ_5_ > Cd_20_MJ_2_._5_ > Cd_10_ > Cd_20_ (Fig. 8).

### Principal component analysis (PCA)

**Fig. 9:**
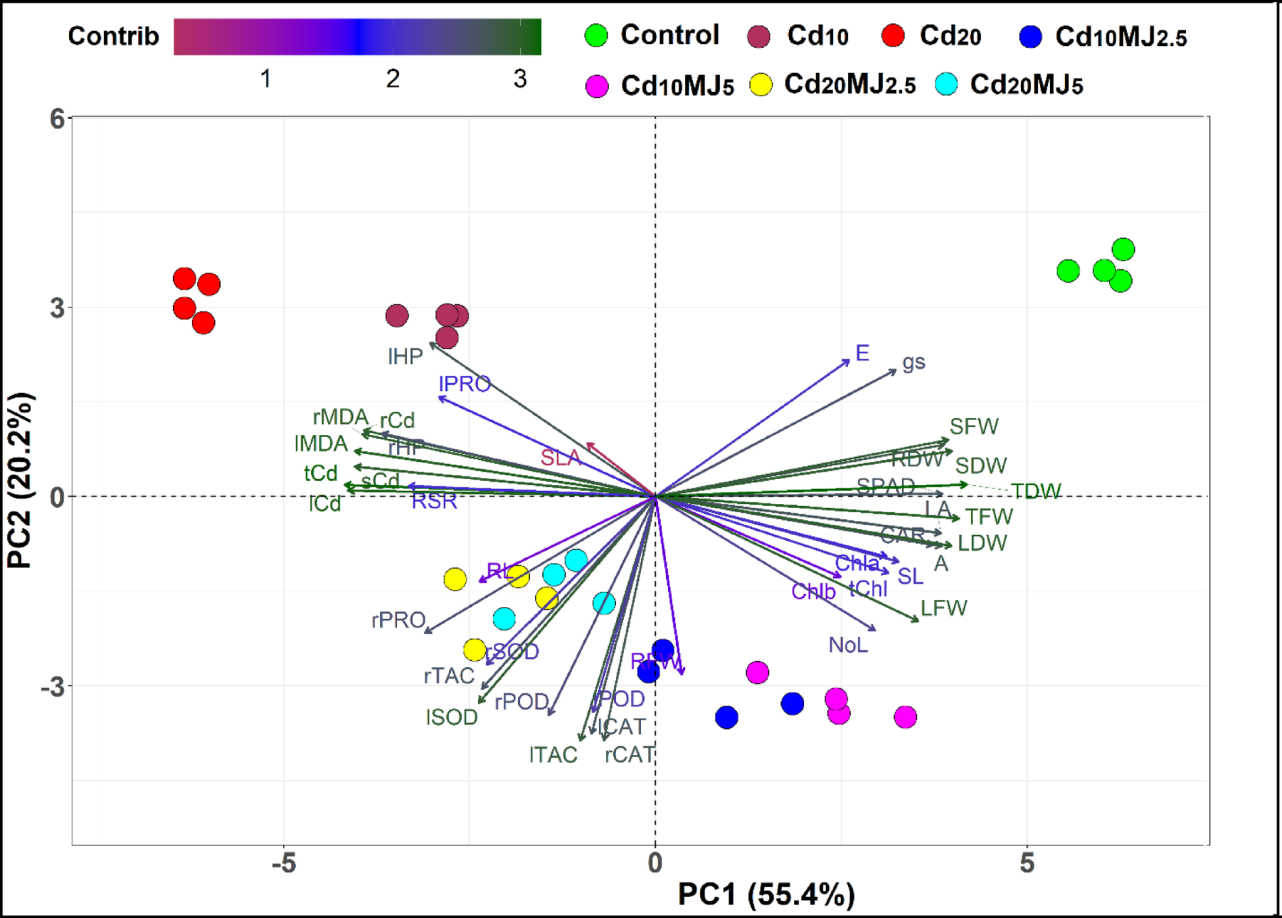
PCA-biplot illustrating the variability of seven growth conditions based on 40 measured traits in water spinach plant. The first and second principal components (PCs) explained 55.4% and 20.2% of the total variability, respectively. The traits’ contribution to the first two PCs is represented by the color gradients and arrow lengths. The longer and darker green arrows denote a higher contributing trait while the shorter and dark red arrows refer to lower contributing traits. Traits description: *r, s, l and t* root, stem, leaf and total, respectively, *PRO* proline, *HP* H_2_O_2_, *Cd* cadmium, *RL* root length, *RSR* root-shoot ratio, *MDA* malonaldehyde, *SLA* Specific leaf area, *g*_*s*_ stomatal conductance, *E* Transpiration rate, *SOD* Superoxide dismutase, *TAC* Total antioxidant capacity, *POD* Peroxidase, *Chl* Chlorophyll, *CAR* Total carotenoids, *NoL* Number of leaf, *LFW* Leaf fresh weight, *A* Rate of photosynthesis, *RFW* Root fresh weight, *RDW* Root dry weight, *CAT* Catalase, *SFW* Stem fresh weight, *SDW* Stem dry weight, *LA* Leaf area, *TFW* Total fresh weight, *SL* Shoot length, *LDW* Leaf dry weight, *TDW* Total dry weight.

**Fig. 10:**
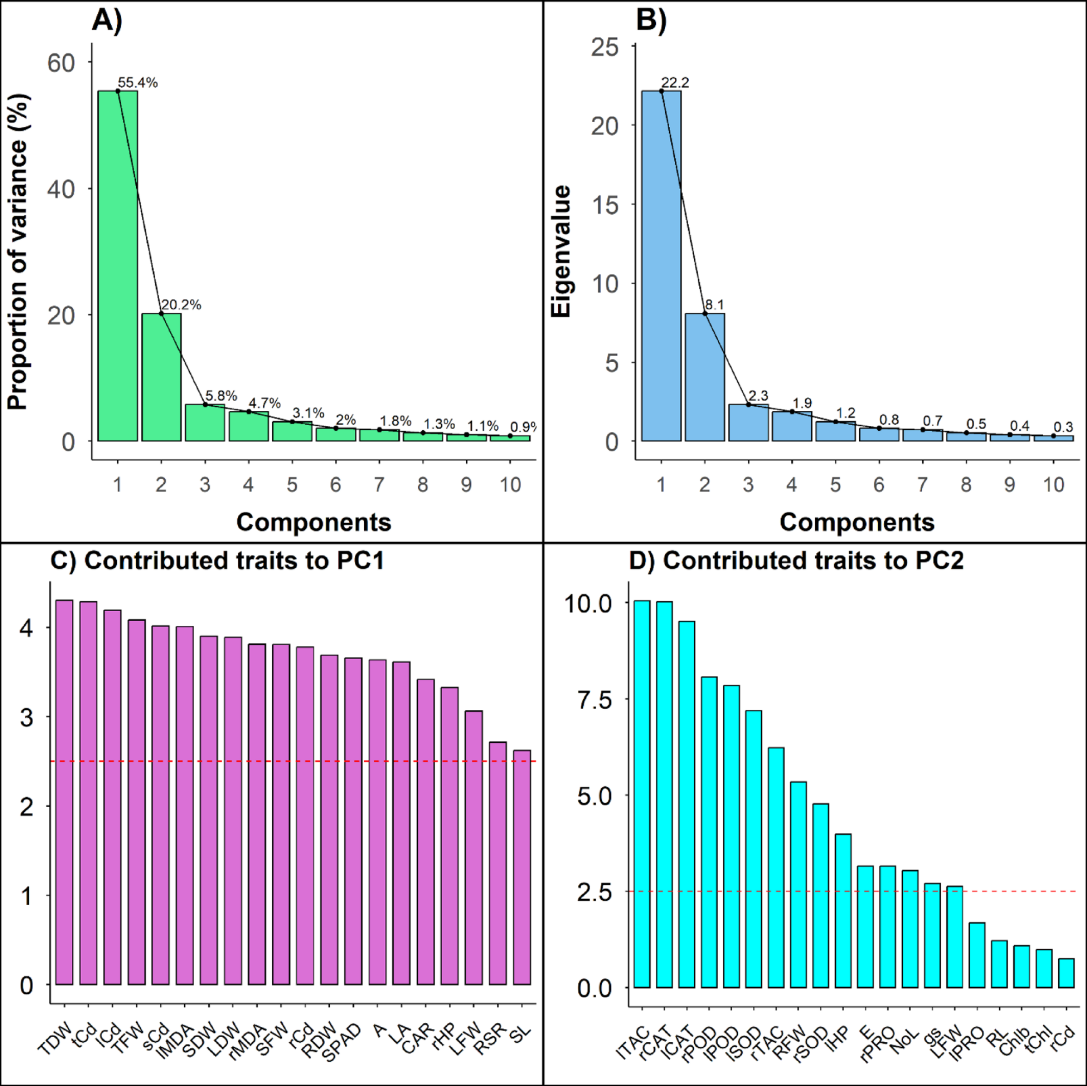
(**A**) Variance proportion (%) and (**B**) Eigenvalues of first 10 principal components (PCs) derived from the PCA-biplot. (**C**) The contribution of the first 20 traits to PC1 and (**D**) First 20 contributing traits to PC2. Bars above the reference lines (red dashed) in each plot are recognized as contributing characters to the respective PC.

The entire dataset of the experiment including forty measured traits was employed to build the principal component analysis (PCA)-biplot to avail prospective relationships among the traits (Fig. 9). The PCA-biplot demonstrates that the top five principal components (PCs) scoring eigenvalues of > 1 accounted for 89.2% of the total variations (Fig. 10A,B). The first two PCs (PC1 and PC2) were considered to construct the PCA-biplot due to their highest percentage (75.2%) of variance (Fig. 10A). The PC1 contributed 55.4% of the total variability among traits and was largely correlated with 23 closely associated traits including TDW, Total Cd content, Leaf Cd content, TFW, Stem Cd content, Leaf LPO, SDW, LDW, Root LPO, SFW, Root Cd content, SPAD, *A*, LA, Root H_2_O_2_ and so on (Figs. 9 and 10C). The PC2 explained 20.2% of the total variability among traits and was mostly linked with 13 traits, particularly the enzymatic antioxidants traits along with RFW, Root Proline, Leaf H_2_O_2,_ *g*_*s*_ and *E* (Fig. 9 and 10D). PCA-biplot showed a distinct separation among the treatments particularly between control and Cd-alone treatments, whereas MeJA applied treatments placed in between control and Cd treatments indicating enhanced tolerance to Cd stress (Fig. 9).

### Correlation analysis

**Fig. 11:**
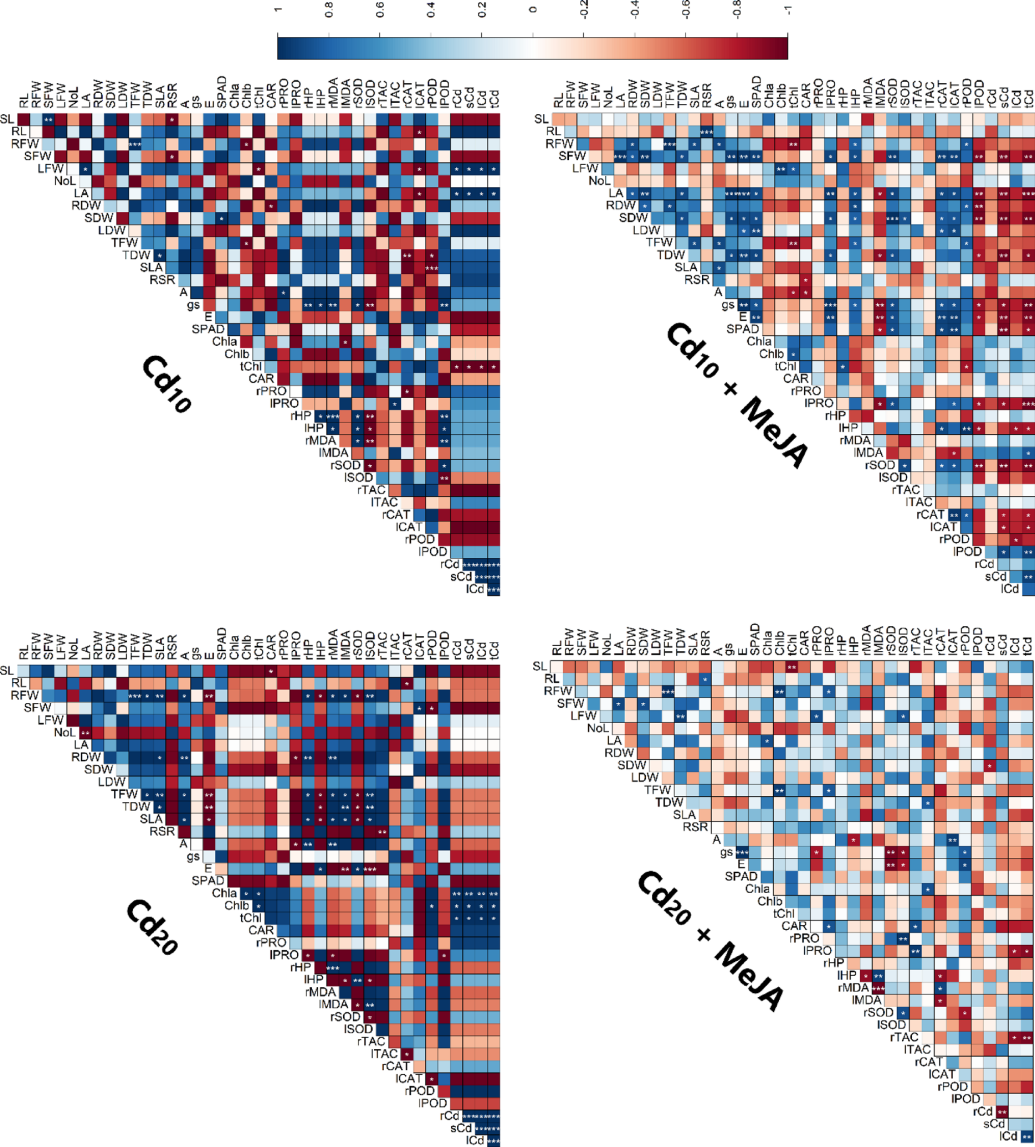
Correlation matrix of studied traits under Cd stress alone (left two panels) and Cd stress + MeJa (right panels). In all matrix plots, red and blue boxes represent positive and negative correlations, respectively, where greater color intensity indicates a higher coefficient. *, **, and *** signify significance at *p* < 0.05, *p* < 0.01, and *p* < 0.001, respectively. Traits description: *r, s, l and t* root, stem, leaf and total, respectively, *PRO* proline, *HP* H_2_O_2_, *Cd* cadmium, *RL* root length, *RSR* root-shoot ratio, *MDA* malonaldehyde, *SLA* specific leaf area, *g*_*s*_ stomatal conductance, *E* transpiration rate, *SOD* superoxide dismutase, *TAC* total antioxidant capacity, *POD* peroxidase, *Chl* chlorophyll, *CAR* total carotenoids, *NoL* number of leaf, *LFW* leaf fresh weight, *A* rate of photosynthesis, *RFW* root fresh weight, *RDW* root dry weight, *CAT* catalase, *SFW* stem fresh weight, *SDW* stem dry weight, *LA* leaf area, *TFW* total fresh weight, *SL* shoot length, *LDW* leaf dry weight, *TDW* total dry weight.

At both 10 and 20 µM Cd concentrations, plants treated with Cd exhibited a notable transfer of Cd from roots to stems and leaves, consequently maintaining a significantly higher correlation coefficient between Root-Cd and stem/leaf-Cd (Fig. 11). The application of MeJA in Cd-treated plants led to a weakening of the relationship between root-Cd and stem/leaf-Cd. At 20 µM Cd, MeJA resulted in a reversal of root- and stem-Cd dynamics, wherein an increase in root Cd led to a significant decrease in its transfer to the stem. Consequently, leaf Cd levels in MeJA-treated plants did not exhibit any positive or negative correlations. With higher Cd doses, there was a decrease in leaf Chl *a*, Chl *b*, and Total Chl levels, possibly due to smaller cell sizes without compromising the overall amount of chloroplasts and chlorophyll content.

## Discussion

An increased application of wastewater, industrial effluents, chemical fertilizers and pesticides to agricultural land particularly in urban and pre-urban areas harshly contaminates and boosts the pools of heavy metals in soils^2,61^. The widespread heavy metal pollution particularly the highly mobile Cd, even at lower concentrations severely impedes plant growth and yield and more consciously, contaminates the food chain threatening human health^62,63^. In recent years, the roles of methyl jasmonate (MeJA) in mitigating heavy metals uptake and toxicity in plants have drawn considerable attention in the scientific community. Therefore, the current study investigated the possible alleviatory role of MeJA on water spinach seedlings to Cd stress contemplating morpho-physiological attributes, oxidative status and Cd accumulation in different plant parts.

Cd exposure in both levels in this study impaired growth of water spinach plants as demonstrated in recent relevant studies with other leafy vegetables^14,16,18–20,64,65^. This Cd-induced cessation of plant growth may be due to the restriction of mitotic cell division of meristematic cells and enlargement of parenchyma tissues damaging the root cells in cortical, endodermis and pericycle regions^66,67^. In addition, Cd may interrupt cell permeability and restrict the absorption of essential ions like N, P, and K retarding plant growth^68^. However, MeJA supplementation with 2.5 and 5 µM levels substantially improved the growth of water spinach plants showing an enhanced tolerance to Cd-toxicity, which is analogous to previous investigations with different vegetables^20,40,42,47,69–72^. Although the longest root length was observed in Cd-treated plants in this study, the root volume was significantly increased in MeJA-treated plants by accelerating lateral root formation. It indicates that MeJA might enhance cell division and facilitate the formation of lateral roots in water spinach plants assisting in greater nutrient uptake. MeJA application under Cd stress displayed increased adventitious root formation in cucumber plant compared to Cd-alone treatment^71^. It was claimed that MeJA activated the cell cycle (promoted transition from G1 to S transition phase) by upregulating the cell cycle-related cyclin-dependent kinase (*CDKs*) genes such as *CycA*,* CycB*,* CDKA* and *CDKB*. Thus, the external application of MeJA rescued the adverse plant growth in water spinach plants, which might be due to replenished cell division and elongation and restraint cell damage. Moreover, MeJA may help in the regulation of other endogenous plant growth-accelerating hormones such as gibberellin and ethylene compensating plant growth under Cd stress^73^. The JA and ABA accumulation were increased due to exogenous MeJA application in okra and improved Cd tolerance by regulating endogenous hormonal metabolism^42^. Modulation of endogenous JA levels has been reported to improve the sensitivity of plants against heavy metal stress in exogenously applied MeJA in tomato^74^ and *Kendelia obovata*^46^ plants.

A direct consequence of suppressed plant growth by Cd is the alterations in photosynthetic pigments and gas exchange parameters^17^ which was apparent in the current study. The decreased photosynthetic pigment contents can be explained by the impaired chloroplast ultrastructure and chlorophyll metabolism, suppressed enzyme activities and perturbed Mg^2+^ and Fe^2+^ uptake that are responsible for pigment biosynthesis and the ROS-induced pigment degradation^15,23^. MeJA application restored the photosynthetic pigments in water spinach leaves under Cd stress in this study with a view of earlier reports that MeJA might have upregulated the gene expression of enzymes involved in pigment synthesis and stimulated nutrient ion uptakes particularly Mg^2+^ and Fe^2+^ and reduced generation of ROS^40,47,75^. The *A* reduction might be resulted due to lower levels of photosynthetic pigments, damage of the light-harvesting complex and the structural and functional disorder of photosystems (I and II)^20,47,76^. Goussi et al.^77^ explained that Cd stress injures the PSII antenna and reduces the efficiency of PSII resulting in impaired electron transport. Moreover, excess Cd ion in leaves declined Rubisco and PEP enzymes activity by altering their structures and by replacing the essential cofactors of carboxylation process such as Mg^2+^ ions and thereby shifting oxygenation reactions or photorespiration^78,79^. Stomatal closure due to Cd exposure may also hinder CO_2_ supply that counteract electron transport chain, thylakoid membranes and photosynthetic enzymes resulting in photosynthetic downregulation. As shown in this study, a linear relationship between the rate of transpiration and inhibition of net photosynthesis is also registered in oilseed, legume and cereal crops under Cd stress with an indication of stomatal closing^80,81^. However, the *A*, *g*_*s*_ and *E* in MeJA-treated leaves in this study were significantly enhanced compared to the plants of Cd-alone treatments. MeJA has been renowned for its ability to protect photosynthetic apparatus, increase Rubisco activity and higher accumulation of photosynthetic pigments improving photosynthesis in plants^30,43^. Per et al.^43^ demonstrated that MeJA prominently alleviated Cd-induced photosynthetic damage through increased S-assimilation and production of reduced glutathione (GSH) in mustard leaves and thus, protected chloroplast structure promoting photosynthetic functions. Several studies have reported an improvement in photosynthesis and stomatal opening by the exogenous application of MeJA under Cd stress in different crop species such as mentha^69^, wheat^82^, faba bean^83^ and rapeseed^84^.

The most detrimental effect of heavy metal stress is the overproduction of ROS (O_2_^−^, H_2_O_2_ and OH^·^) which subsequently damages nucleic acids, proteins, lipids and cellular pigments in plants^15,17,21,25^. In this current investigation, Cd notably increased the MDA and H_2_O_2_ contents in root and leaves of water spinach plants with a clear indication of enhanced oxidative damage to the plants and this perturbation was considerably retrieved by the application of MeJA. Cd reduces cell proliferation and differentiation by damaging DNA, RNA and enzymatic proteins responsible for cell-repairing processes^85^. Gill and Tuteja^86^ reported cell death in tobacco under Cd stress due to the buildup of NADPH-oxidase in peroxisomes and the overproduction of H_2_O_2_ in fatty acids. The peroxidation of membrane polyunsaturated fatty acid leads to MDA accumulation, which is considered a marker of lipid peroxidation that disintegrates membrane fluidity, inflates electrolytic leakages, hinders enzymes’ activity and interferes with protein channeling in plants^87,88^. Likewise, earlier studies also confirmed the protective role of MeJA to oxidative damage under Cd stress exhibiting a significant reduction of MDA and H_2_O_2_ contents in other crops like rapeseed^84^, tomato^40^, pigeon pea^72^, pea^47^, mustard^43^, *Kandelia obovata*^46^ and rice^41^.

Plants possess an active defense system against oxidative damage due to Cd stress through prompt synthesis of potential enzymatic and non-enzymatic antioxidants that effectively scavenge the harmful ROS^89,90^. Proline is a non-essential amino acid that has been reported to be increased upon Cd stress in many plant species^17,83^ including the current study. Proline accumulation in plants is acclaimed as an approach to counteract Cd stress through the adjustment of osmotic potential and membrane structure stabilization and reduction of oxidative damage^91–93^. In this study, a significantly lower accumulation of proline was recorded in MeJA-treated plants compared to Cd treatments. The lesser synthesis of proline in MeJA-treated plants could be due to the lower production of MDA and H_2_O_2_ and reduced pressure of oxidative stress. Under Cd stress, drastic upsurge of diverse enzymatic antioxidants such as superoxide peroxidase (SOD), catalase (CAT), ascorbate peroxidase (APX), peroxidase (POD), glutathione reductase (GR), dehydro-ascorbate reductase (DHAR) and monodehydroascorbate reductase (MDHAR) were reported in many crop plants^15,65,94,95^. Hong et al.^96^ highlighted that the Cd-mediated increased activity of SOD and APX enzymes is regulated by the overexpression of *ZmWRKY4* gene located at the nucleus of the mesophyll protoplast in maize. The findings of this study revealed that exogenous MeJA boosted the activities of SOD, CAT and POD enzymes and total antioxidant activity (TAC) compared to individual Cd treated plants. It indicates an extent level of antioxidative defense through the increased expression of genes related to these enzymatic antioxidants by MeJA and thereby maintained cellular redox equilibrium protecting the plants from cell membrane damage^34,39^. MeJA as a signalling molecule has been shown to improve heavy metal tolerance in *Brassica napus* by upregulating the gene expression of key antioxidants and secondary metabolites such as phenylalanine ammonia-lyase (*PAL*), polyphenol peroxidase (*PPO*), cinnamyl alcohol dehydrogenase (*CAD*) and lipoxygenase genes^37^. MeJA supplementation substantially elevated the activities of SOD, CAT and GR enzymes promoting AsA-GSH cycle for rapid scavenging of H_2_O_2_, thereby reduced MDA levels in Cd-treated wheat seedlings^82^. MeJA exposure increased the reduced GSH and sulfur (S)-assimilation and therefore enhanced Cd tolerance in mustard plants preventing the membrane structure and functions^43^. JA increased the transcriptional activity of GSH biosynthesis associated genes which are vital components of antioxidative defense mechanism to regulate heavy metal-induced oxidative damage^36^. The enhanced activity of CAT and the components of AsA-GSH cycle were involved in successful scavenging of H_2_O_2_ reducing oxidative damage in MeJA-treated okra plants under Cd stress^42^. MeJA application improved Cd-toxicity in *Cajanus cajan* plants by minimizing oxidative damage through the modulation of AsA-GSH cycle, calcium and *MAPK* (mitogen-activated protein kinase) signalling^72^. Several transporter protein gene families include ATP-binding cassette transporter (*ABC*), iron-regulated transporter-like protein (*ZIP*), cation diffusion facilitator (*CDF*) and natural resistance associated macrophage protein (*NRAMP*) are involved in heavy metal transport in the plant body^97^. MeJA could play a crucial role by downregulating the expression of these transporter genes attenuating Cd toxicity in plants. MeJA alleviated Cd-toxicity in pigeon pea plants by involving in the calcium and kinase signalling pathways and boosted the expression of *CALM*, *IP3*, *CDPK2* and *MPKs* genes and downregulated the expression of *IRT1* and *HMA3* (metal transporters) genes^72^.

Excess Cd in the growth medium accelerated Cd uptake by the water spinach plants in this study reflecting higher Cd content in root, stem and leaf. However, Cd contents in root, stem and leaf were considerably reduced due to MeJA supplementation. Heavy metals in soil inhibit the uptake of essential minerals and can easily be accessed in the root cells via different cation (Ca^2+^, Zn^2+^, Fe^2+^, Mn^2+^, Mg^2+^) transporters^40,78,98^. MeJA could help in facilitating ion uptakes and depressing the Cd^2+^ influxes in the roots and thereby decreasing Cd accumulation in plant parts, particularly in the root. The plant cell wall serves as the first obstacle of Cd uptake and its translocation to the cytoplasm shielding the protoplast against Cd stress. The polysaccharides and proteins of the cell wall contain negatively charged functional hydroxyl, amino, carboxyl and aldehyde groups that bind and limit the transmembrane translocation of heavy metal ions into the cytoplasm maintaining normal functions of the plant cell^99–101^. Wei et al.^40^ reported that MeJA enriches the binding, precipitation and crystallization capabilities of the cell wall depositing higher Cd in cell wall than cytoplasm in root. They suggested that MeJA regulated the synthesis of cell wall polysaccharides and negatively charged Cd-binding groups maintaining higher Cd in root cell wall and limited Cd translocation. The cytoplasm also contains sulfur-rich ligands that interact with Cd and preserved the Cd-complexes into the vacuole and functioning as a second line defense in plant^102^. Few gene families such as metallothioneins (*MTs*) and phytochelatin synthases (*PCs*) became activated under Cd stress and involved in uptake, xylem/phloem loading and sequestration of heavy metals^97,98^. Exogenous application of MeJA downregulated the expression of *MT1* and *KoMT2* genes responsible for Cd uptake and translocation and enhanced Cd tolerance in pigeon pea and *Kendelia obovata* plants^46,72^. The expression of the *TAGS1* and *TaPCS1* genes encoding GSH and *PCs* were increased by MeJA application reflecting an enhanced tolerance in wheat plants to Cd stress^103^. The application of MeJA regulated the transcript levels of *PCS1*, *PCS2*, *ABCC1* and heavy metal-transporting *P1B ATPases* (*HMAs*) genes and decreased Pb translocation from roots to the aerial parts of rice^104^. Therefore, the reduced Cd uptake, higher root Cd contents and its lower translocation to the aerial parts of water spinach plant due to MeJA application in this study might be linked with the stimulated translocation barriers and accelerated Cd-sequestration by the root cell wall and cytoplasmic vacuoles along with the upregulation at the transcriptional levels. Thus, MeJA-oriented restrained Cd in the cell wall and cytoplasm may reduce the Cd content in important cell organelles such as chloroplast, mitochondria and endoplasmic reticulum enabling the plant’s normal physiological and biochemical functions and thereby, improved tolerance in water spinach plants to Cd toxicity^39^. As leafy vegetables are directly consumed by humans, the reduced levels of Cd in shoots through exogenous application of MeJA would therefore potentially reduce the Cd-mediated health risk.

The relative changes of 40 measured traits in Cd-treated plants compared to control were illustrated in this study by multivariate data analysis such as hierarchical clustering heatmap, PCA-biplot and correlation matrix. This has been done to obtain a clear view of the plant responses to Cd exposure and their extent of Cd tolerance with exogenous MeJA application. Results revealed that 5 µM MeJA application was more effective in mitigating Cd toxicity than 2.5 µM in both levels (10 and 20 µM) of Cd exposure in water spinach plants. PCA identified several traits that could be considered as selection criteria for crop improvement to Cd tolerance. The most important selection traits for Cd tolerance were TDW, partitioned Cd accumulation, lipid peroxidation and antioxidative enzymes (SOD, CAT and POD) activities. The findings of this study provided useful information regarding optimization of exogenous MeJA doses for effective remediation of Cd-induced morpho-physiological disorders in water spinach plants under hydroponic system. However, for large or small-scale farming of water spinach in Cd-polluted field soils, further field trial in Cd-contaminated areas is necessary for validating the beneficial effects of the studied phytohormones with the concentration levels used in this study. Moreover, the effective way of exogenous application such as seed priming and foliar spraying need to be properly investigated including cost–benefit ratio. Prior to farmers’ field trial, pot experiments with synthetic Cd-polluted soils are also crucial to ratify the sole effect of Cd as the contaminated field soils may contain other toxic heavy metals.

## Conclusions

Cd-mediated impaired growth and physiology, induced oxidative damage and increased Cd uptake and translocation in water spinach plants were effectively retrieved by the exogenous application of methyl jasmonate (MeJA). Exogenous MeJA application with 5 µM concentration over 2.5 µM performed better in alleviating the toxic effects of Cd. However, further studies will be required to test the effects of MeJA (dose-responsive and ways of exogenous application) in water spinach plants grown particularly in Cd-contaminated soils and areas. To develop a Cd-tolerant water spinach cultivar through a future breeding program, a few traits such as total dry matter, photosynthetic rate, Cd contents, lipid peroxidation and enzymatic antioxidant activity could be considered as selection criteria.

## Data Availability

The datasets generated during and/or analysed during the current study are available from the corresponding author (Md. Sabibul Haque, mshaqcb@bau.edu.bd) on reasonable request.
